# Genomic imprinting in human placentation

**DOI:** 10.1002/rmb2.12490

**Published:** 2022-12-01

**Authors:** Eri H. Kobayashi, Shun Shibata, Akira Oike, Norio Kobayashi, Hirotaka Hamada, Hiroaki Okae, Takahiro Arima

**Affiliations:** ^1^ Department of Informative Genetics Tohoku University School of Medicine Sendai Japan

**Keywords:** complete hydatidiform mole (CHM), ES‐TS transdifferentiation, genomic imprinting (GI), human placenta, trophoblast stem (TS) cells

## Abstract

**Background:**

Genomic imprinting (GI) is a mammalian‐specific epigenetic phenomenon that has been implicated in the evolution of the placenta in mammals.

**Methods:**

Embryo transfer procedures and trophoblast stem (TS) cells were used to re‐examine mouse placenta‐specific GI genes. For the analysis of human GI genes, cytotrophoblast cells isolated from human placental tissues were used. Using human TS cells, the biological roles of human GI genes were examined.

**Main findings:**

(1) Many previously identified mouse GI genes were likely to be falsely identified due to contaminating maternal cells. (2) Human placenta‐specific GI genes were comprehensively determined, highlighting incomplete erasure of germline DNA methylation in the human placenta. (3) Human TS cells retained normal GI patterns. (4) Complete hydatidiform mole‐derived TS cells were characterized by aberrant GI and enhanced trophoblastic proliferation. The maternally expressed imprinted gene p57KIP2 may be responsible for the enhanced proliferation. (5) The primate‐specific microRNA cluster on chromosome 19, which is a placenta‐specific GI gene, is essential for self‐renewal and differentiation of human TS cells.

**Conclusion:**

Genomic imprinting plays diverse and important roles in human placentation. Experimental analyses using TS cells suggest that the GI maintenance is necessary for normal placental development in humans.

## 1 INTRODUCTION

**FIGURE 1:**
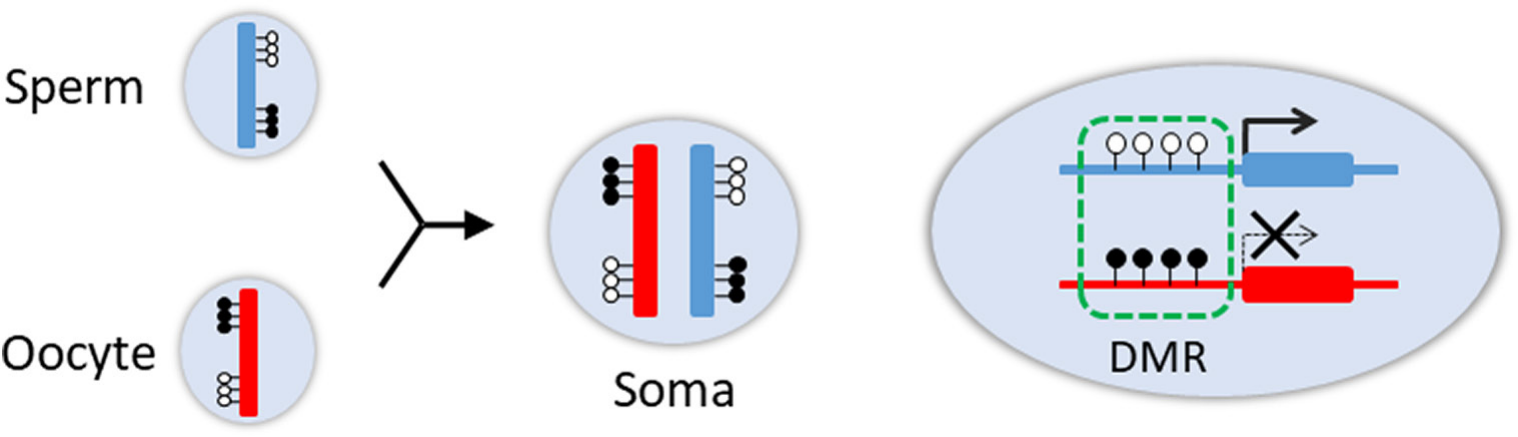
Regulation of GI gene expression. DNA methylation of DMRs is acquired during germ cell formation and is stably maintained in the somatic cell as a genomic imprinting memory. Black and white circles indicate methylated and unmethylated CpGs, respectively.

Genomic imprinting (GI) is an epigenetic phenomenon that describes parent‐of‐origin patterns of monoallelic gene expression reported in mammals.^1^ This differential gene expression is initiated within the germline when discrete regions of the genome acquire DNA methylation in one germline but not the other. These differentially methylated regions (DMRs), present within most imprinted loci, are key to establishing and, in some cases, maintaining imprinted gene expression (Figure 1). The difficulty in producing parthenogenetic embryos is due to the fact that mammals must have both male and female GI‐bearing genomes in order to develop normally.^2^

In primordial germ cells, which differentiate into the germ cells that produce the next generation, most of the memory of the epigenome from both parents, including GI, is erased. A new methylated GI is then established in DMRs in the premeiotic spermatogonia of the male during the fetal period and in the maturing oocytes of the female, where the ovum begins to mature for ovulation. This DMR methylation is protected against global demethylation immediately after fertilization and is maintained stably throughout life in subsequent somatic cells.^3^, ^4^, ^5^, ^6^, ^7^, ^8^

We now know that there are over 100 genes in mammals that are regulated by genomic imprinting and many of these have critically important roles in early development and metabolic and behavioral processes after birth.^9^, ^10^ The importance of normal GI in humans is best illustrated by a number of rare but striking childhood developmental disorders associated with imprinted loci such as Beckwith–Wiedemann syndrome (OMIM 130650), Angelman syndrome (OMIM 105830), Silver–Russell syndrome (OMIM 180860), and Prader–Willi syndrome (OMIM 176270), and childhood cancers such as retinoblastoma^11^ (https://doi.org/10.1002/path.890).

Genomic imprinting is seemingly a disadvantage for the survival of mammals because if the active allele is mutated, the inactive allele cannot compensate for it. Various hypotheses have been made regarding the biological significance of this GI phenomenon in mammals, such as the parthenogenesis prevention theory, the malignant ovarian tumor prevention theory, the genetic‐conflict hypothesis, and the exogenous virus protection theory.^12^, ^13^, ^14^, ^15^, ^16^, ^17^ Considering that GI is unique to mammals with placentas, it is also thought that GI in mammals acquiring and evolving the placental organ is related to the formation of the “placentation hypothesis”.^18^ The placenta regulates the transport of nutrients, gases, and waste products between the maternal and fetal blood circulation and plays a critical role in fetal growth and development.^19^, ^20^ Thus, it has been suggested that decreased placental function not only causes low birth weight in infants and affects the postnatal physical and neuropsychiatric development of infants but also increases the risk of cancer and lifestyle‐related diseases later in life.^21^ This review focuses on our findings on the functional importance of GI in the mouse and human placenta.

## 2 GENOMIC IMPRINTING GENES IN THE MOUSE PLACENTA

Both gynogenetic and androgenetic embryos in mice are lethal during the second trimester of pregnancy.^22^, ^23^ However, their phenotypes are very different. Gynogenetic embryos show little placental formation, whereas androgenetic embryos show severe fetal growth restriction with relatively normal placentas. Approximately 150 GI genes have been found in mice and humans (https://www.geneimprint.com/site/home), many of which are expressed in the placenta. Furthermore, analysis using knockout (KO) mice has led to the discovery of important functions of GI genes in placental development.^3^, ^4^ For example, KO mice of the GI genes *Ascl2* and *Peg10* fail to form part of the cell layer that makes up the placenta, which is lethal during mid‐embryogenesis.^24^, ^25^ In both of these KO mice, replacement of the placenta using a technique called tetraploid embryo complementation produces a viable individual. These facts suggest that *Ascl2* and *Peg10* function in a placenta‐specific manner during mouse development.

A number of placenta‐ and brain‐specific GI genes have been reported in mice.^26^, ^27^, ^28^, ^29^ Of these, most of the mouse placenta‐specific GI genes were expressed from the maternal genome, and only a few were expressed from the paternal genome. The placenta is inevitably contaminated with a small amount of maternal tissue; maternal uterine decidua tissues and blood cells are present in spongiotrophoblast and labyrinth layers^30^, ^31^, ^32^ and the ectoplacental cone is already invaded by maternal blood at embryonic day (E) 6.5.^27^ We re‐investigated the placenta‐specific GI genes comprehensively by embryo transfer to a surrogate mother in another strain of mice to avoid contamination of maternal tissue.^33^ The results showed that many of the placenta‐specific GI genes reported so far had been incorrectly identified as being imprinted due to maternal tissue contamination, and only 11 genes (nine genes of maternal expression and two genes of paternal expression) were confirmed to be imprinted (Table 1). In addition, GI of these 11 genes was also reconfirmed using mouse trophectoderm stem (TS) cells with DNA polymorphisms.

**TABLE 1:** List of mouse placenta‐specific GI genes

Imprinting status in the mouse				Imprinting status in the human placenta
Chr.	Gene	Placenta	TS cells	
2	*Sfmbt2*	NA	Imprinted	–
6	*Tfpi2*	–	Imprinted	Imprinted (polymorphic)
6	*Ppp1r9a*	Imprinted	Imprinted	Imprinted (polymorphic)
7	*Th*	Imprinted	ND	NA
7	*Ascl2*	Imprinted	Imprinted	–
7	*Tspan32*	–	Imprinted	–
7	*Tssc4*	Imprinted	Imprinted	–
7	*Ano1*	–	Imprinted	Imprinted (polymorphic)
8	*Gab1*	Imprinted	Imprinted	–
17	*Slc22a3*	Imprinted	Imprinted	Imprinted (polymorphic)
17	*Slc22a2*	Imprinted	ND	Imprinted (polymorphic)

Animal cloning by somatic cell nuclear transfer (SCNT) provides a unique model for understanding the mechanisms of nuclear epigenetic reprogramming to a state of totipotency.^34^, ^35^ However, regardless of the species or donor cells, the cloning efficiency is very low and the incidence of phenotypic abnormalities in offspring is frequent.^34^, ^35^ SCNT‐derived mice are typically characterized by placental hypertrophy.^36^ Because SCNT produces individuals without passing through germ cells, the GI pattern observed in somatic cells is stably maintained in cloned mice with the exception of Xist.^37^, ^38^, ^39^, ^40^, ^41^, ^42^ However, it was unclear whether placenta‐specific GI genes exhibit normal GI patterns in the placenta of cloned mice.^36^ We found that uniparental expression of placenta‐specific GI genes was disrupted in SCNT‐derived placentas, demonstrating that SCNT cannot restore placenta‐specific GI.^43^ Abnormal expression of placenta‐specific GI genes has been proved to be responsible for placental hypertrophy.^44^, ^45^ However, none of the human homologs of mouse placenta‐specific GI genes were imprinted in the human placenta, and the overall picture of GI in the human placenta was still unknown.^33^

## 3 HUMAN PLACENTA‐SPECIFIC GI GENES

**FIGURE 2:**
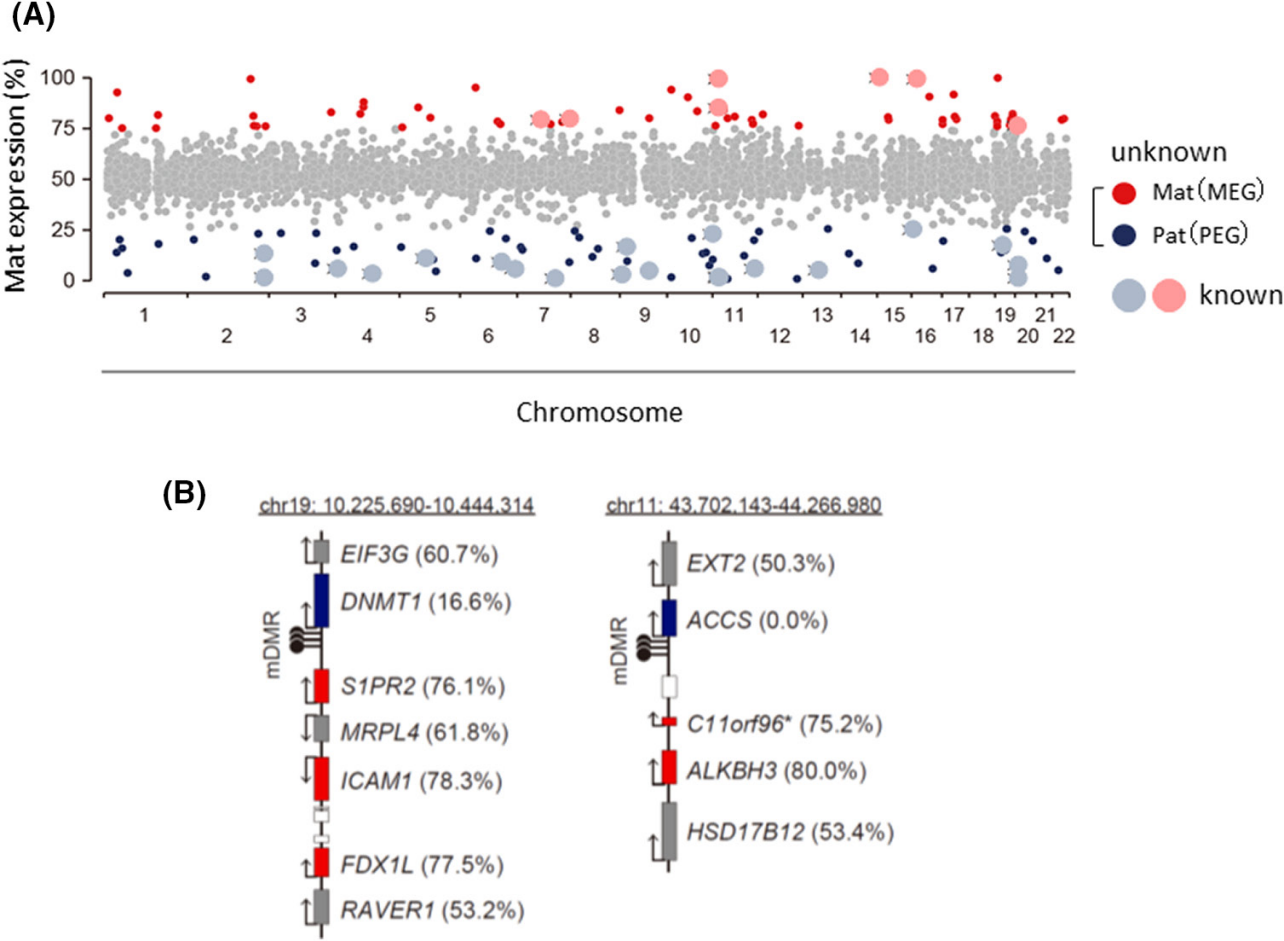
Human placenta‐specific imprinted gene map. (A) Chromosomal distribution of placenta‐specific GI genes. The *x*‐axis and *y*‐axis show chromosome numbers and the maternal expression ratio ([Mat‐expression] ratio), respectively. Each *x* indicates a known or unknown GI gene with a [Mat‐expression] ratio >70% or <30%. The other genes are shown in gray. (B) The *DNMT1* and *ACCS* imprinted gene clusters. Genes with maternal and paternal expression are shown in red and blue, respectively. The other genes are shown in gray. Genes without available allelic expression ratios are shown as white boxes without gene symbols.^46^

To comprehensively identify human GI genes, we obtained and purified undifferentiated cytotrophoblast (CT) cells from placental tissue in the first trimester. Then, we analyzed allele‐specific gene expression using RNA sequencing.^46^ As a result, it was found that GI genes were present on various chromosomes in a total of 110 genes, 53 paternal, and 57 maternal allele‐dominant expressions (Figure 2), and the number of human placenta‐specific GI genes was approximately 10 times more than that of mice. We found that multiple GI genes form clusters regulated by single DMRs.

Most GI genes are regulated by the DNA methylation of DMRs acquired during germ cell formation.^3^, ^47^ Approximately 25 DMRs have been identified in mice.^48^ We identified the involvement of DMRs in the regulation of the expression of the human placenta‐specific GI genes by genome‐wide DNA methylation profiling of human oocytes, spermatozoa, blastocysts, and placental CT cells.^46^, ^49^ In humans, the number of DMRs was higher than that in mice, and the placenta retained a large number of DMRs. Many of these DMRs are maternally methylated, and demethylation of the maternal genome was found to be incomplete in humans.^50^, ^51^, ^52^ However, the novel human placenta‐specific GI genes found by our group and other groups have not been reported as being imprinted in mice, highlighting the low conservation of placental GI.

Among the human placenta‐specific GI genes, CUL7 encodes an E3 ubiquitin ligase scaffold protein, which has been reported to be hypomethylated in the promoter region and to show increased expression in placentas with fetal growth restriction.^53^ Deficiency of CUL7 also causes 3‐M syndrome type I, a congenital anomaly syndrome with fetal growth restriction, severe postnatal growth restriction, and characteristic facial features.^54^ In addition, CUL7 deficiency in mice causes vascular abnormalities in the decidua and these mice exhibit phenotypes such as impaired placental development and fetal growth restriction.^53^ These observations suggest that CUL7 plays an important role in human placentation. CYP2J2, another gene identified as a novel placenta‐specific imprint gene, encodes one of the cytochromes, p450, and is known as a drug‐metabolizing enzyme. CYP2J2 is highly expressed in placentas in women with hypertensive disorders of pregnancy and is presumed to be one of the genes involved in the pathogenesis.^55^ In addition, elevated metabolites of CYP2J2 are also observed in preeclampsia model rats, and these metabolites might also be involved in the pathogenesis.

The reason why these human placenta‐specific GI genes are not subject to GI regulation in the mouse placenta is currently unknown. However, there should be a unique epigenetic mechanism for the regulation of DNA methylation in placental cells that differs from that in fetal cells.^56^, ^57^, ^58^ The poor conservation of placental GI might be related to differences in placental structure, gestational periods, and litter size. Comparative analysis of placental GI in more animal species may provide new insights into the necessity of placenta‐specific GI genes in placental evolution.

## 4 HUMAN TS CELLS AND GENOMIC IMPRINTING

**FIGURE 3:**
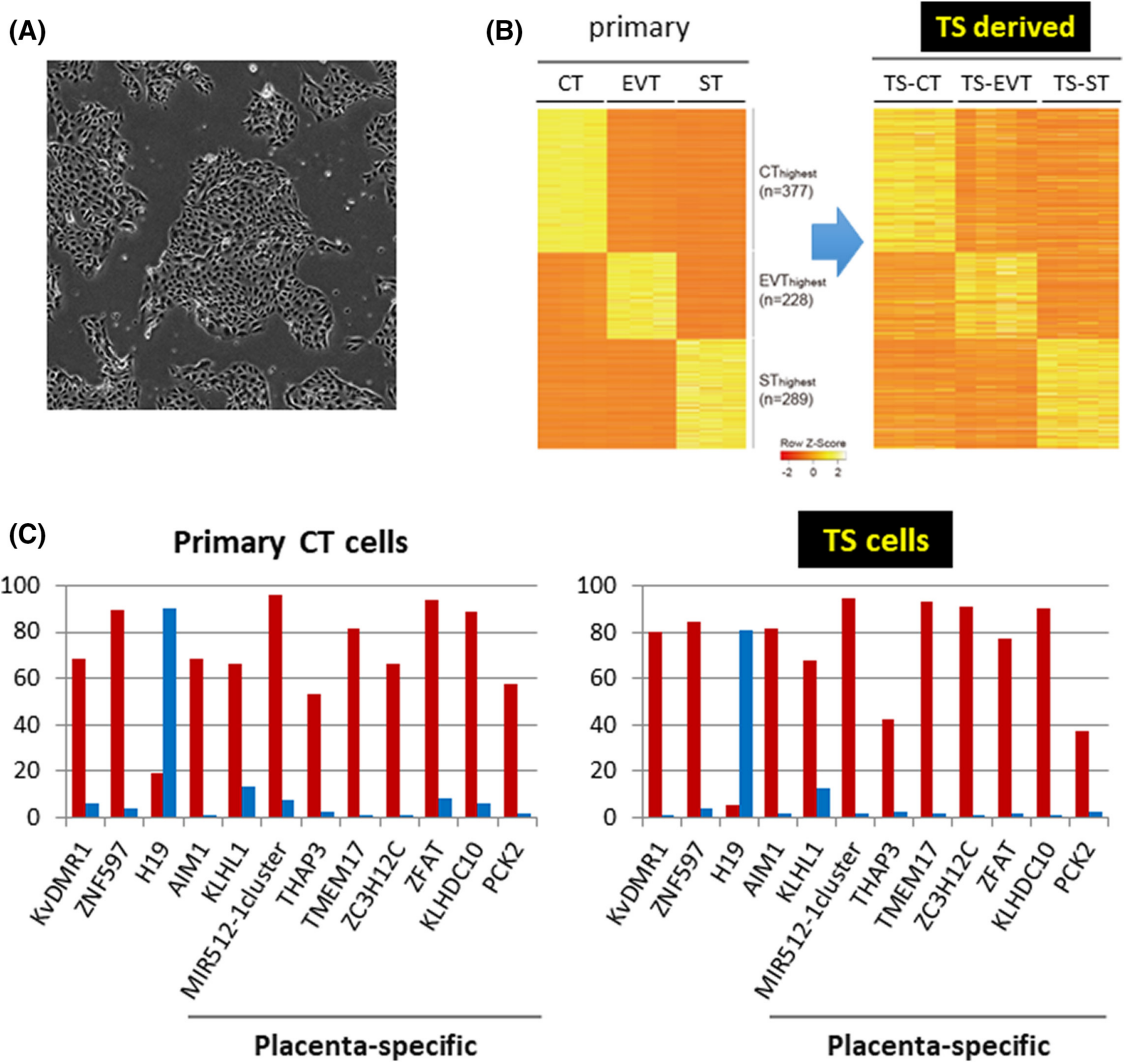
Characteristics of human TS cells. (A) Morphology of human TS cells. (B) Heatmap representation of genes predominantly expressed in CT, EVT, or ST cells. The predominant gene expression in CT, EVT, or ST cells (left) show similar expression patterns in TS cells and their derivatives (right). All gene expression levels are normalized by z‐score. (C) Allele‐specific methylation regions of placenta‐specific GI genes (%) in primary CT and TS cells. DNA methylation levels of the maternal and paternal allele are shown in red and blue, respectively.^60^

Conventional studies on human trophoblast cells have utilized primary cultured trophoblast cells, choriocarcinoma cells, immortalized cells, etc. However, primary cultured trophoblast cells are difficult to maintain and expand in vitro, and choriocarcinoma and immortalized cells do not reflect the properties of normal trophoblast cells. Therefore, it was difficult to accurately evaluate the function and regulatory mechanisms of GI genes in the human placenta. Many researchers believed that human TS cells, if established, would be a powerful tool to understand human trophoblast development and functions. Mouse TS cells were first derived from blastocysts and the extra‐embryonic ectoderm of post‐implantation embryos. In 1998, Tanaka et al. found that in the presence of FGF4, mouse TS cells self‐renew indefinitely without losing their ability to differentiate into all trophoblast lineages,^59^ and approximately 20 years later, we succeeded in establishing human TS cells (Figure 3A).^60^ Human TS cells were able to proliferate and remained undifferentiated for more than 80 passages in a culture medium containing a Wnt activator, EGF, and inhibitors of TGF‐β, histone deacetylase (HDAC), and Rho‐associated protein kinase (ROCK). TS cells can differentiate into syncytiotrophoblast (ST) cells, which mediate gas and nutrient exchange and produce placental hormones, and extravillous trophoblast (EVT) cells, which remodel spiral arteries in the endometrium.^61^, ^62^ We found that the gene expression of human TS cells and their derivatives were closely related to those of primary trophoblast cells (Figure 3B).

In somatic cells, most genomic regions except for the promoter and enhancer regions are hypermethylated, whereas in human trophoblast cells, approximately 40% of the genome is partially methylated.^58^ Also, some promoter regions are hypermethylated in somatic cells but hypomethylated in human trophoblast cells.^63^, ^64^ These features including allele‐specific methylation status of placenta‐specific GI genes were also maintained in human TS cells. (Figure 3C).

To analyze how human TS cells function in vivo, we subcutaneously injected TS cells into NOD/SCID‐immunodeficient mice. Immunostaining of the trophoblast marker KRT7 revealed that the injected cells invaded the dermal and subcutaneous tissues. SDC1‐positive ST‐like cells and HLA‐G‐positive EVT‐like cells were observed in the peripheral region. We also found that the host mouse serum contained a large amount of hCG. Interestingly, we found that some of the ST‐like cells contained blood‐filled lacunae, reminiscent of primitive ST cells that form during implantation, invade the maternal endometrium, and erode maternal sinusoids.^62^ Thus, we recapitulated some important features of human implantation using human TS cells.

Human TS cells exhibit genetic and functional properties similar to those of human trophoblast cells and may be useful for understanding GI and the pathogenesis of developmental disorders with trophoblast defects, such as miscarriage, preeclampsia, and intrauterine growth restriction.

## 5 COMPLETE HYDATIDIFORM MOLE DERIVED TS CELLS

A complete hydatidiform mole (CHM) is a gestational trophoblastic disease characterized by enhanced trophoblast proliferation, swollen villi, and the absence of embryonic component. Approximately 15%–20% of CHM cases are followed by malignant gestational trophoblastic neoplasms, including invasive moles and choriocarcinoma.^65^, ^66^, ^67^ Since CHM is androgenetic in origin,^68^, ^69^ aberrant GI gene expression should play a major role in the pathogenesis of CHM. However, it was not clear which imprinted gene(s) are involved in the pathogenesis of CHM.

**FIGURE 4:**
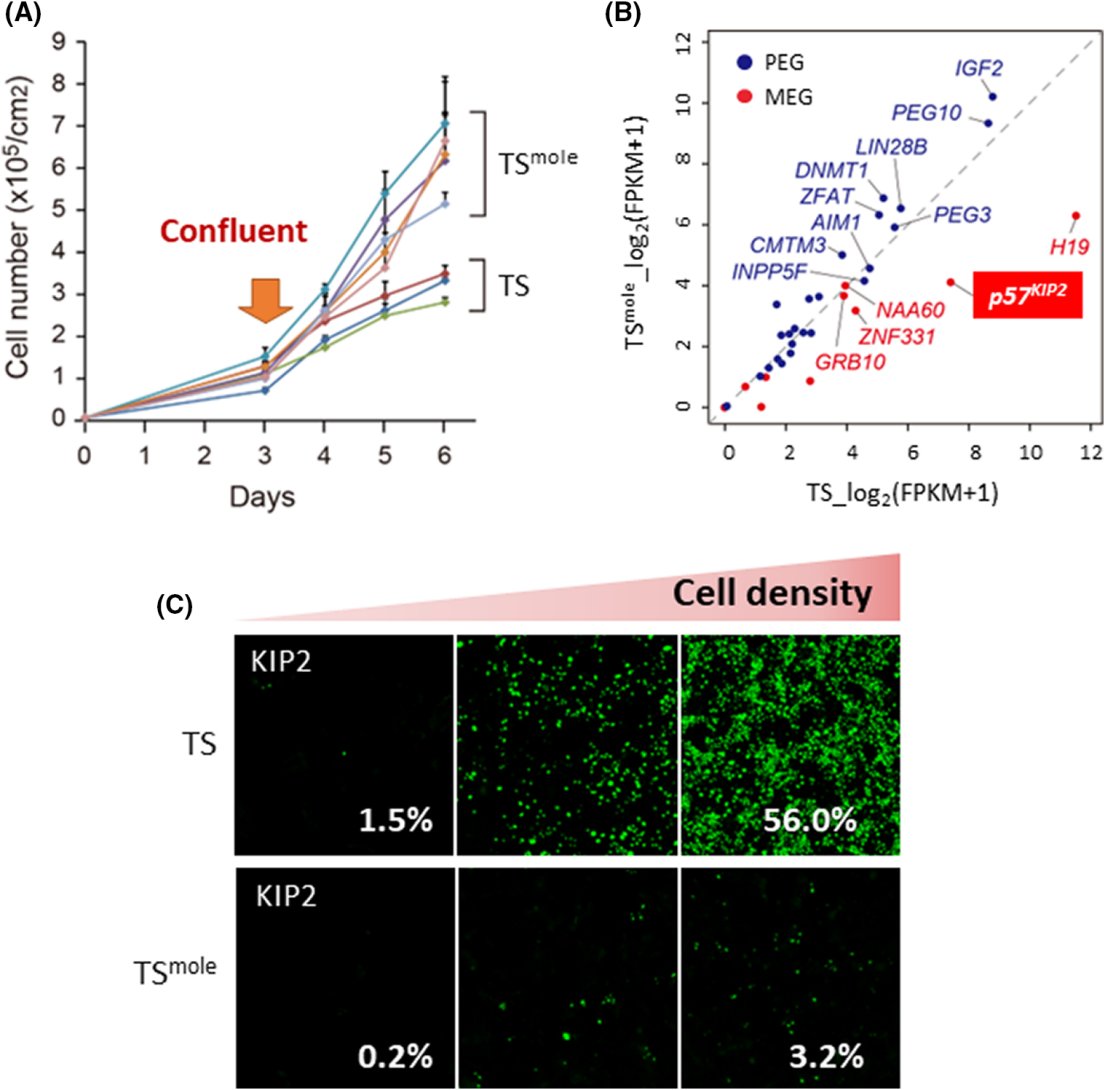
Characteristics of TS^mole^ cells. (A) TS^mole^ cells' ability to proliferate. TS^mole^ cells continue to proliferate after cell density reaches confluent. TS^mole^ cells have significantly higher cell numbers than TS at day 6 (*p* < 0.01; Student's *t* test). Data are shown as means ± SDs. (B) Expression levels of GI genes in TS and TS^mole^ cells. Maternally and paternally expressed genes are shown in red and blue, respectively. (C) Cell density‐dependent induction of p57KIP2 in TS cells but not in TS^mole^. p57KIP2 is stained with Alexa Fluor 488 (green). White number indicates the percentage of p57KIP2‐positive cells.^70^

We purified trophoblast cells from placental tissue of CHM and established TS cell lines (Figure 4A).^70^ Overall, normal TS cells from healthy individuals and CHM‐derived TS cells exhibited similar gene expression patterns. However, the expression of GI genes was exceptionally different. In CHM‐derived TS cells, the expression of the paternally expressed imprinted gene tended to increase, while the expression of the maternally expressed imprinted gene tended to decrease (Figure 4B). In addition, DMRs showed sperm‐derived methylation patterns.

Abnormal trophoblast proliferation is observed in CHM, suggesting that CHM‐derived TS cells might have a higher cell proliferation rate than normal TS cells. Interestingly, the proliferative capacity of CHM‐derived TS cells was enhanced compared with that of normal TS cells only when they were cultured at high densities. Thus, CHM‐derived TS cells were found to be resistant to cell cycle arrest by contact inhibition (Figure 4C). In normal TS cells, the expression of p57KIP2 (CDKN1C), an imprinted gene showing maternal expression, increased in a cell density‐dependent manner, but in CHM‐derived TS cells, p57KIP2 did not increase even at high‐cell densities. p57KIP2 expression leads to cell cycle arrest by inhibiting cyclin‐dependent kinases.^71^, ^72^, ^73^ Consistently, forced expression of p57KIP2 in CHM‐derived TS cells resulted in cell cycle arrest, and TS cells with p57KIP2 knockout were resistant to cell cycle arrest induced by contact inhibition. Therefore, we revealed that the decreased expression of p57KIP2 is involved in the abnormal proliferation observed in CHM‐derived TS cells.

## 6 PERINATAL DISORDERS AND IMPRINTING ABNORMALITIES

It is well known that congenital disorders such as Beckwith–Wiedemann syndrome, Angelman syndrome, Silver–Russell syndrome, and Prader–Willi syndrome are caused by abnormal expression patterns of GI genes. Some of these congenital disorders may also involve abnormalities of the placenta. Beckwith–Wiedemann syndrome has three main symptoms of intravesical hernia, macrosomia, and increases in placental weight, while polyhydramnios is also observed.^74^ Fetuses with Beckwith–Wiedemann syndrome, in the presence of a mutation in p57KIP2, cause preeclampsia in the mother at a high frequency.^75^ Consistently, loss of p57KIP2 also causes preeclampsia‐like symptoms in mice.^76^ Transient neonatal diabetes and Kagami–Ogata syndrome (uniparental disomy (14) pat), which include abnormalities in GI genes, also show placental hypertrophy.^77^, ^78^ Conversely, placental hypoplasia is seen in some patients with Silver–Russell syndrome.^79^ These findings are in good agreement with the phenotype of KO mice.^80^, ^81^ It is thought that overexpression of IGF2 may cause placental overgrowth and its decreased expression causes growth suppression of the placenta.^82^, ^83^, ^84^ Overexpression of the retrotransposon‐derived gene RTL1 causes symptoms of paternal disomy of chromosome 14 (Kagami–Ogata syndrome), and loss of expression causes symptoms of maternal disomy.^85^ Paternal disomy of chromosome 14 results in placental overgrowth, which is consistent with the phenotype of mice overexpressing Rtl1.^85^, ^86^ With some exceptions, many maternally expressed genes negatively regulate placental growth, whereas paternally expressed genes positively regulate placental growth. This is in good agreement with findings in mouse gynogenetic and androgenetic embryos.^22^, ^23^ Small for gestational age, fetal growth restriction, and HDP are also frequently associated with imprinting abnormalities.^87^ Preeclampsia has also been reported to be associated with abnormalities in imprinted microRNAs.^88^, ^89^

## 7 EFFECTS OF ASSISTED REPRODUCTIVE TECHNOLOGY (ART) ON GI

Assisted reproductive technology (ART) has become increasingly common due to later marriage and improvements in medical technology in developed countries. Major epigenetic events including the establishment of GI take place in germ cells and preimplantation embryos, and these cells are very vulnerable to environmental changes.^90^, ^91^ ART procedures perform multiple manipulations of gametes and embryos during critical periods of epigenetic reprogramming, suggesting a link between ART and abnormal epigenetic modifications.^92^, ^93^, ^94^, ^95^, ^96^ Previous studies suggest that children born after ART have higher rates of imprinting and psychiatric disorders than naturally conceived children.^97^ ART is also expected to be associated with the development of Small‐for‐Gestational‐Age and HDP.^98^ Moreover, in adults, ART might have an impact on the incidence of lifestyle‐related diseases such as cancer, hypertension, and diabetes.^99^

It is not yet clear whether ART or the infertile patients' own genetic background is really responsible for the increase in the number of patients mentioned above. There is ongoing debate about the potential for ART procedures and processes to alter the development of gametes, embryos, and fetuses. Large‐scale, long‐term follow‐up studies are needed to further investigate the impact of ART.

## 8 ROLE OF GI IN TRANSDIFFERENTIATION OF ES CELLS INTO TROPHOBLAST CELL LINEAGES

In mammals, the first cell differentiation after fertilization is the specification of the inner cell mass (ICM) and the trophectoderm (TE).^100^ The ICM is the future fetus and yolk sac, and the TE is the placenta. In humans and other primates, however, the details of how this cell fate specification is regulated are still unclear. Human ES cells have properties similar to post‐implantation epiblast and are classified as primed.^101^, ^102^ The primed form can also be “reset” to a naive form similar to the preimplantation ICM under specific culture conditions.^103^, ^104^, ^105^, ^106^ A recent study reported that naive human ES cells, unlike those of mice, have the ability to differentiate into extraembryonic lineages.^107^, ^108^ It has been reported that primed human ES cells also differentiate into trophoblast lineages by treatment with BMP4.^109^ However, considering that epiblast cannot differentiate into trophoblast lineages after implantation in vivo, it is controversial whether primed ES cells have the ability to differentiate into trophoblast cells.^110^, ^111^, ^112^

**FIGURE 5:**
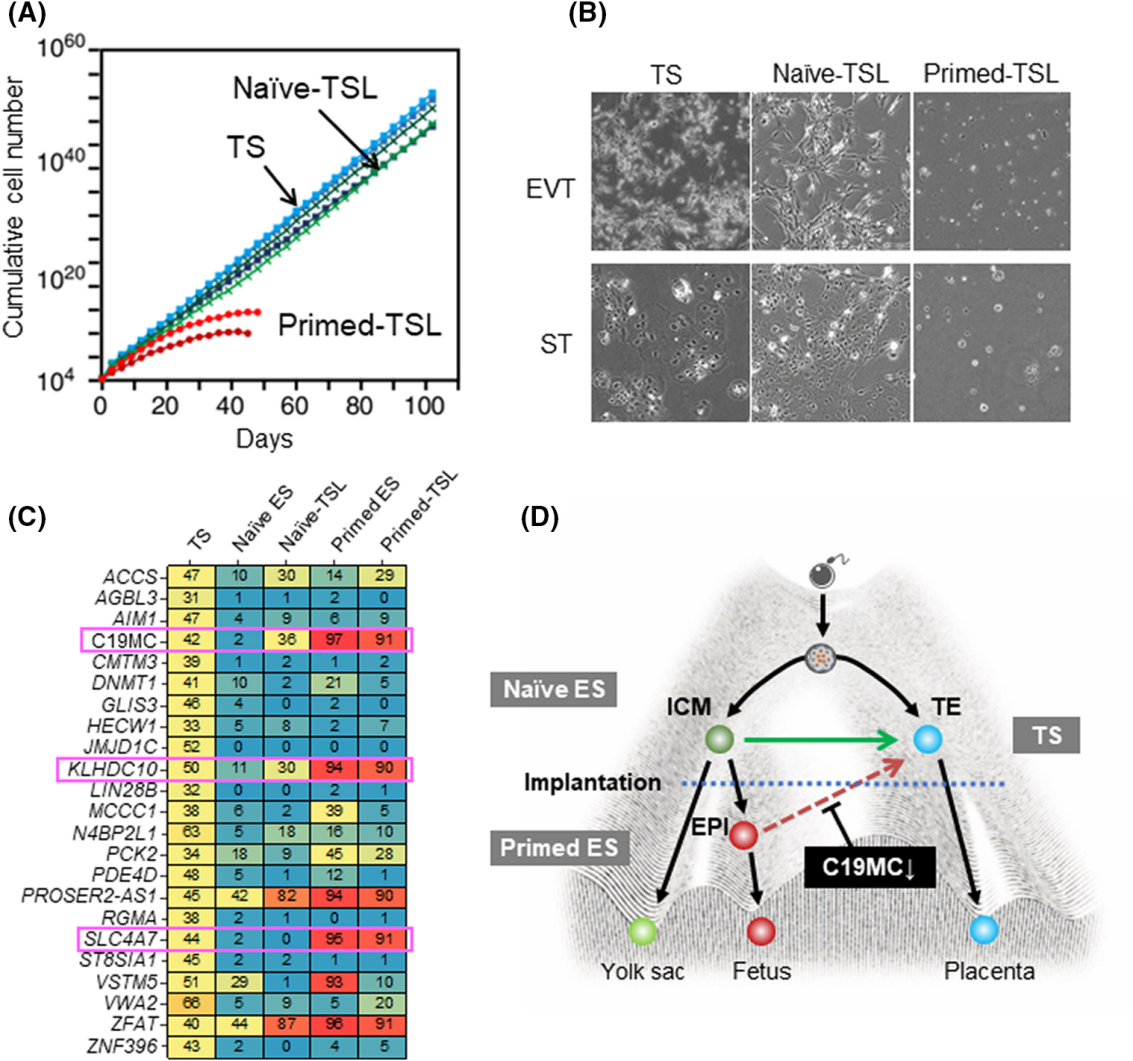
Characteristics of TSL cells derived from naive and primed ES cells. (A) Cell proliferation potential. (B) Cell differentiation potential. (C) DNA methylation levels of placenta‐specific GI genes in ES, TS, and TSL cells. Three placenta‐specific GI genes that were hypermethylated in primed TSL cells but not in naive TSL and TS cells are indicated by pink boxes. (D) Summary of transdifferentiation of ES cells into TS cells.^113^

We transdifferentiated primed and naive human ES cells into TS‐like (TSL) cells and analyzed their proliferation and differentiation ability and epigenomic status in detail.^113^ Primed TSL cells, which were TSL cells transdifferentiated from primed ES cells, had a lower proliferative capacity than TS cells, and their ability to differentiate into EVT and ST cells, which is essential for the function of the placenta, was also incomplete. Naive TSL cells had a proliferative potential similar to TS cells and retained the differentiation potential (Figure 5A,B). In an exhaustive analysis of gene expression and DNA methylation to determine the differences between primed and naive TSL cells, both cells showed generally similar gene expression and methylation patterns, but also some differences. In particular, we found that some placenta‐specific GI genes were highly methylated and markedly decreased in expression in primed TSL cells, unlike in TS cells and naive TSL cells (Figure 5C). Of these genes, we focused on the C19MC region. C19MC is located on human chromosome 19 and is a large primate‐specific microRNA (miRNA) cluster of approximately 100 kb, containing 46 miRNAs.^114^, ^115^ C19MC miRNA expression is restricted to the placenta, and only the paternal allele, which is regulated by an upstream DMR,^116^ is active. When the expression of C19MC miRNAs was analyzed by miRNA‐seq, almost all of them showed high expression in naive TSL cells but markedly low expression in primed TSL cells. When the CRISPR/Cas system was used to KO C19MC in TS cells to model primed TSL cells, the proliferation and differentiation capacities of the KO TS cells were decreased, suggesting that the expression of C19MC is essential for the proliferation and differentiation of TS cells. In addition, primed ES cells expressing C19MC were produced by inducing demethylation of the C19MC DMR^117^ using the dCas‐TET system. These cells successfully differentiated into TSL cells that retained properties almost identical to TS cells. Thus, it was clarified that the primate‐specific miRNA cluster C19MC is important for proliferation and differentiation of TS cells (Figure 5D). These results not only explain the differences in differentiation potentials between naive and primed human ES cells but also provide a good example of the regulation of cell fate specification by GI genes during embryonic development in humans.

## 9 CONCLUSION

In this review, the basic knowledge of GI and the human placenta is summarized based on data from previous studies. As we have outlined, imprinting abnormalities can lead to a variety of diseases, and ART may affect the state of imprinting. Studies using human TS cells have also revealed some of the functions of imprinted genes experimentally. Derivation of human TS cells from placentas of patients with pregnancy complications is expected to reveal pathologies associated with epigenomic mutations. In the future, human TS cells may be applied to clinical research such as development of treatments for pregnancy complications and disease prevention in children.

## FUNDING INFORMATION

Japan Society for the Promotion of Science Grants‐in‐Aid for Scientific Research (JSPS KAKENHI) grants 21H04834, 21H03072, and 19H05757, Japan Agency for Medical Research and Development (AMED) grants JP22gm1310001.

## CONFLICT OF INTEREST

Authors have no conflict of interest to be declared.

## DISCLOSURES

Our derivation of human TS cells was approved by the Institutional Review Board (IRB) at Tohoku university (2014–1‐879).
